# Cationic chitosan-propolis nanoparticles alter the zeta potential of *S*. *epidermidis*, inhibit biofilm formation by modulating gene expression and exhibit synergism with antibiotics

**DOI:** 10.1371/journal.pone.0213079

**Published:** 2019-02-28

**Authors:** Teik Hwa Ong, Ebenezer Chitra, Srinivasan Ramamurthy, Catherine Chong Sze Ling, Stephen Periathamby Ambu, Fabian Davamani

**Affiliations:** 1 School of Postgraduate Studies, International Medical University, Kuala Lumpur, Malaysia; 2 Division of Applied Biomedical Sciences and Biotechnology, School of Health Sciences, International Medical University, Kuala Lumpur, Malaysia; 3 School of Pharmacy, International Medical University, Kuala Lumpur, Malaysia; Central University of Tamil Nadu, INDIA

## Abstract

*Staphylococcus epidermidis*, is a common microflora of human body that can cause opportunistic infections associated with indwelling devices. It is resistant to multiple antibiotics necessitating the need for naturally occurring antibacterial agents. Malaysian propolis, a natural product obtained from beehives exhibits antimicrobial and antibiofilm properties. Chitosan-propolis nanoparticles (CPNP) were prepared using Malaysian propolis and tested for their effect against *S*. *epidermidis*. The cationic nanoparticles depicted a zeta potential of +40 and increased the net electric charge (zeta potential) of *S*. *epidermidis* from -17 to -11 mV in a concentration-dependent manner whereas, ethanol (Eth) and ethyl acetate (EA) extracts of propolis further decreased the zeta potential from -17 to -20 mV. Confocal laser scanning microscopy (CLSM) depicted that CPNP effectively disrupted biofilm formation by *S*. *epidermidis* and decreased viability to ~25% compared to Eth and EA with viability of ~60–70%. CPNP was more effective in reducing the viability of both planktonic as well as biofilm bacteria compared to Eth and EA. At 100 μg/mL concentration, CPNP decreased the survival of biofilm bacteria by ~70% compared to Eth or EA extracts which decreased viability by only 40%-50%. The morphology of bacterial biofilm examined by scanning electron microscopy depicted partial disruption of biofilm by Eth and EA extracts and significant disruption by CPNP reducing bacterial number in the biofilm by ~90%. Real time quantitative PCR analysis of gene expression in treated bacteria showed that genes involved in intercellular adhesion such as *IcaABCD*, *embp* and other related genes were significantly downregulated by CPNP. In addition to having a direct inhibitory effect on the survival of *S*. *epidermidis*, CPNP showed synergism with the antibiotics rifampicin, ciprofloxacin, vancomycin and doxycycline suggestive of effective treatment regimens. This would help decrease antibiotic treatment dose by at least 4-fold in combination therapies thereby opening up ways of tackling antibiotic resistance in bacteria.

## Introduction

*Staphylococcus epidermidis* survives on the skin as normal flora under the epithelium and is recognized as an opportunistic pathogen commonly encountered in hospital-acquired infections without critical implications. The bacteria attach to solid surfaces forming biofilms; this process is characterized by distinct phases–initiation of establishment and colonization leading to infectious stage, primary reversible adhesion developing into secondary irreversible adhesion, and biofilm formation [1]. *S*. *epidermidis* can adhere to both biotic and abiotic surfaces of indwelling or implanted medical devices or tissues and form biofilms, leading to treatment failure and relapse of infections [2]. It is implicated in cardiac prosthetic valve infections causing endocarditis, which might lead to intra-cardiac abscesses and mortality. *S*. *epidermidis* exhibits resistance to β-lactam antibiotics, which are commonly used in clinical settings [3]. Its antimicrobial resistance is mainly linked to its capability to colonize and produce biofilms.

Bacteria residing in biofilms are resistant to antibiotic treatments and escape the host immune responses. Biofilm formation is a multifarious process that is regulated by various genes, whose exact function in each step of biofilm formation remains poorly understood [2]. At sub-inhibitory concentrations of antibiotics, *S*. *epidermidis* was found to induce biofilm formation by upregulating the expression of biofilm-related genes such as *ica*, *sarA* and *embp* [3–5]. Discovery of natural therapeutic agents with the capability to prevent biofilm formation or inhibit pre-formed biofilms without contributing to bacterial resistance would be ideal to combat biofilm-related infections.

Antibiotics overuse/misuse has been blamed for the emergence of antibiotic resistance, resulting in the search for better alternatives for treatment. There is an increasing interest in combination therapy involving natural products with antibacterial properties synergistically working with conventional antibiotic therapy. This results in improved clinical outcomes as the dose of antibiotics required for effective treatment can be reduced and the undesirable side effects of drugs can be further minimized.

Propolis is a resinous substance collected by honeybees from various floral sources including flowers, pollen and buds. The common composition of propolis is as follows; 50% plant resin and balsam, 30% wax, 10% essential and aromatic oils, 5% pollen and 5% various other constituents [6]. The chemical components of propolis are flavonoids, phenolic and aromatic compounds, which can vary depending on the geographical regions, type of bees involved and floral sources [7]. Propolis is reported to retain a broad spectrum of pharmacological activities such as antibacterial [8, 9], antiviral [10], antifungal [11], anti-inflammatory [12] and antioxidant properties [13].

Bulgarian propolis showed synergistic effect when combined with chloramphenicol, tetracycline and neomycin, and has proven effective for the treatment of *Salmonella typhi* infection [14]. Synergistic interactions between propolis and several antibiotics including streptomycin, cloxacillin and cefixime have also been reported [15, 16]. Malaysian propolis nanoformulation with chitosan has been reported to be effective against *Enterococcus faecalis* biofilms [17].

In this study, the anti-bacterial property of Malaysian propolis nanoformulation against *S*. *epidermidis* biofilms and its effectiveness in combination therapy with antibiotics are investigated. This study focuses on the effect of chitosan-propolis nanoparticles on bacterial surface charge, biofilm formation and their ability to alter the expression of genes involved in biofilm formation.

## Materials and methods

### Bacterial strain and culture

*Staphylococcus epidermidis* strain (ATCC 14990) was used as a standard strain in this study. *S*. *epidermidis* was cultured at 37°C in tryptic soy broth supplemented with 1% glucose in a rotary incubator (LM-510, YIHDER, Taiwan). Bacterial suspension of 0.5 McFarland units was standardized to be used as inoculum for the experiments. All experiments were carried out in triplicates with three independent repeats.

### Zeta potential of *S*. *epidermidis*

Ethanol (Eth) and ethyl acetate (EA) extracts of propolis as well as chitosan-propolis nanoparticles (CPNP) [17] were added to the bacterial suspension and incubated for 1 hour at 37°C and the membrane zeta potential of bacterial cells was measured with Zetasizer Nano Zs (Malvern Instruments, UK) [18]. Untreated bacteria were used as negative control. The zeta potential of propolis Eth and EA extracts and CPNP was also measured. The experiments were carried out in triplicates with three independent repeats.

### Confocal laser scanning microscopy analysis of live/dead bacteria

Biofilm formation was initiated in Fluorodish glass bottom culture dishes (World Precision Instruments, Sarasota, FL) and incubated for 48 hours at 37°C with/without propolis Eth or EA extracts or CPNP. After incubation, the dishes were gently rinsed with saline and stained using LIVE/DEAD kit (Invitrogen Molecular Probes, USA). The samples were stained with 3 μL each of SYTO9 and propidium iodide for 15 minutes in the dark. Untreated biofilms were used as negative control. The biofilms were observed using a LEICA TCS SPE Confocal Microscope (Leica Microsystems, Wetzlar, Germany) at Universiti Teknologi MARA, Malaysia. The images were generated using Leica LAS AF software and three-dimensional plots of biofilm samples were constructed with ImageJ software. The experiments were carried out in triplicates with three independent repeats.

### Determination of efficacy of antimicrobial treatments

Bacterial suspensions were inoculated in 24-well plates (Eppendorf, Hamburg, Germany) and treated with varied concentrations of Eth or EA extracts of propolis or CPNP and incubated at 37°C in a rotary incubator for biofilm formation. Untreated bacteria were used as negative control. After 24 hours, the plates were gently washed with saline to remove the planktonic bacteria and the bacterial biofilm was dislodged the by gentle pipetting. The supernatant planktonic bacteria and biofilm bacteria were serially diluted and plated on tryptic soy agar and the CFU in each was determined. The experiments were carried out in triplicates with three independent repeats.

### CPNP-antibiotics synergy test

Broth microdilution assay was used to evaluate the minimum inhibitory concentration (MIC) [19] of antibiotics and CPNP against *S*. *epidermidis*. The antibiotics (rifampicin, ciprofloxacin, vancomycin, doxycycline and gentamicin) were purchased from Sigma-Aldrich (St. Louis, MO, USA). For checkerboard microdilution assay, the individual antibiotic was titrated across the x axis of a 96-well plate while CPNP were titrated across the y axis. Titration was performed by two-fold serial dilution in Mueller-Hinton broth. Untreated negative controls were included. Bacterial suspensions were added to each well to a final volume of 200 μL and incubated at 37°C for 24 hours. The experiments were carried out in triplicates with three independent repeats.

Synergy was evaluated by calculating the fractional inhibitory concentration index (FICI).

$$FICI=\left(\frac{MIC\;of\;antibiotic\;in\;combination}{MIC\;of\;antibiotic\;alone}\right)+\left(\frac{MIC\;of\;CPNP\;in\;combination}{MIC\;of\;CPNP\;alone}\right)$$

Synergy was defined as FICI ≤ 0.5, no interaction was defined as FICI > 0.5–4, and antagonism was defined as FICI >4. The inhibitory concentration is inferred from the absence of bacterial growth in the wells.

### Biofilm imaging by scanning electron microscopy

Glass coverslips were placed in 6-well plates, bacterial suspension was added to the wells and incubated at 37°C in a rotary incubator for 24 hours to facilitate biofilm formation. The bacteria were treated with propolis Eth or EA extracts or CPNP. Untreated control was included. After incubation, the glass coverslips were rinsed with saline followed by fixation with 2.5% glutaraldehyde. Biofilms were serially dehydrated, air-dried and sputter coated with gold (SC7620 Mini Sputter Coater, Quorum Technologies, UK). The biofilms formed were observed using a TM3000 tabletop scanning electron microscope (Hitachi, Japan). The experiments were carried out in triplicates with three independent repeats.

### Biofilm-related gene expression analysis by real-time QPCR

Bacteria collected from biofilm bacteria and planktonic bacteria with/without treatment were centrifuged for 10 minutes at 5000 g. RNeasy mini kit (Qiagen) was used to extract RNA from biofilm bacteria and planktonic bacteria following the manufacturer’s instructions. Total RNA was converted to cDNA using Superscript Vilo master mix (Invitrogen, USA). QPCR analysis was performed using 25 ng of cDNA and 1.25 μM of the appropriate primers using qPCRBio SYGreen master mix (PCR Biosystems, UK) and iQ5 real-time PCR detection system (Bio-Rad Laboratories, Hercules, California, USA). The genes involved in the formation/regulation of biofilms (*rsbU*, *sarA*, *icaA*, *icaB*, *icaC* and *icaD*), adhesin genes (*embp* and *atlE*) were analyzed and a house keeping gene *tpi* was used as control. The sequences of the primers used are listed in S1 Table. The experiments were carried out in triplicates with three independent repeats.

### Statistical analysis

All quantitative results are presented as mean ± standard error. Statistical comparisons of QPCR data groups were analyzed by one-way ANOVA and followed by post-hoc Tukey’s honest significant difference (HSD). Comparisons between treatments were considered significant when P value was < 0.05.

## Results

### Alteration of S. epidermidis zeta potential by propolis and CPNP

**Fig 1:**
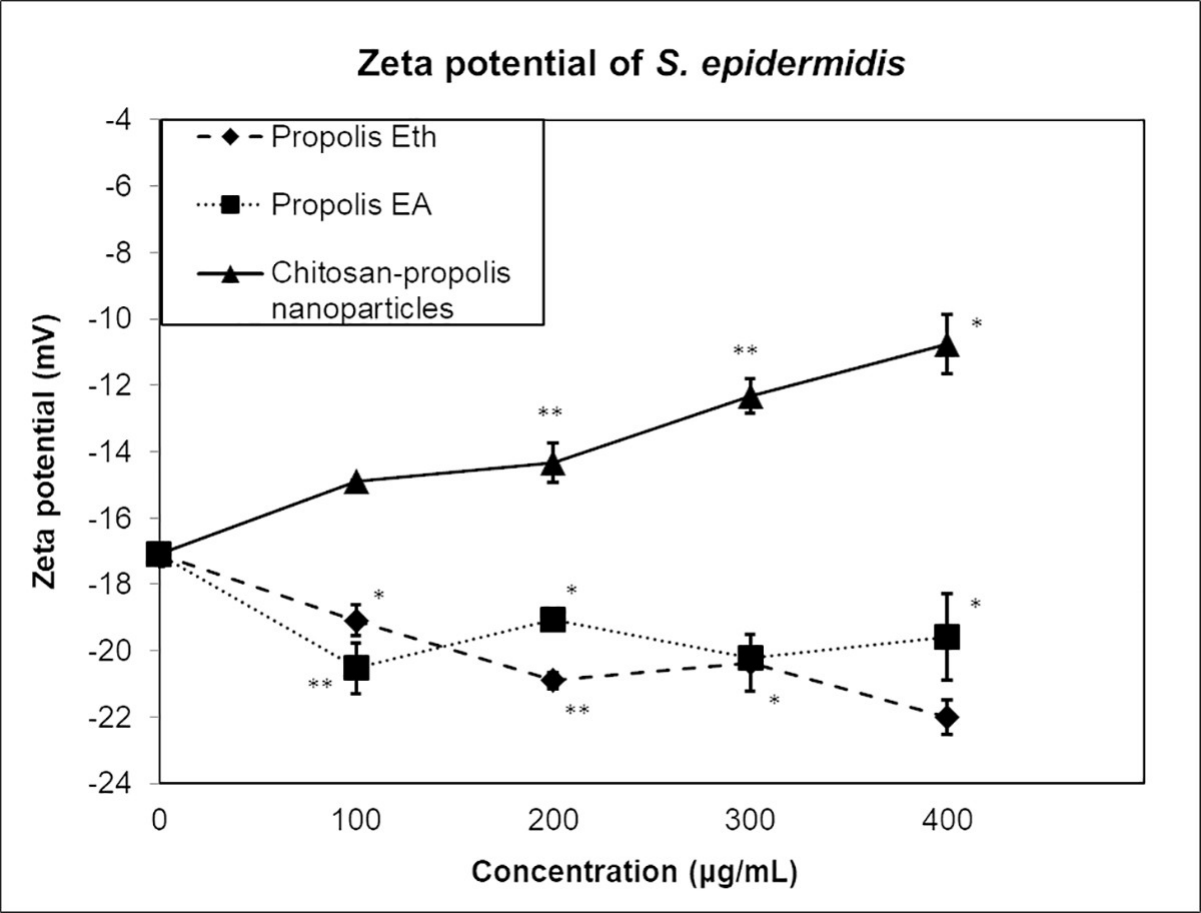
Zeta potential of *S*. *epidermidis* treated with propolis extracts or CPNP. Graph representing the zeta potential of *S*. *epidermidis* treated with different concentrations of propolis Eth (◆) or propolis EA (▀) or chitosan-propolis nanoparticles (▲). The data point at the concentration “0” depicts untreated control. Abbreviations: Eth: ethanol; EA: ethyl acetate.

The zeta potential of untreated *S*. *epidermidis* was found to be -17.1 mV when measured using a zetasizer (Fig 1), indicating a negative cell surface charge. Propolis Eth and EA extracts measured alone had a negative charge of -2.69 mV and -2.78 mV respectively. Therefore, *S*. *epidermidis* treated with propolis Eth and EA extracts had a further reduction in the zeta potential to about -20 mV. In contrast, CPNP measured alone displayed a positive zeta potential of +40 mV due to the cationic property of the nanoparticles. Treatment of *S*. *epidermidis* with CPNP caused the zeta potential of the treated bacteria to increase. With increasing concentration of CPNP, zeta potential of the bacteria increased to about -11 mV as depicted in Fig 1.

### Confocal scanning laser microscopy (CLSM) analysis for bacterial viability

**Fig 2:**
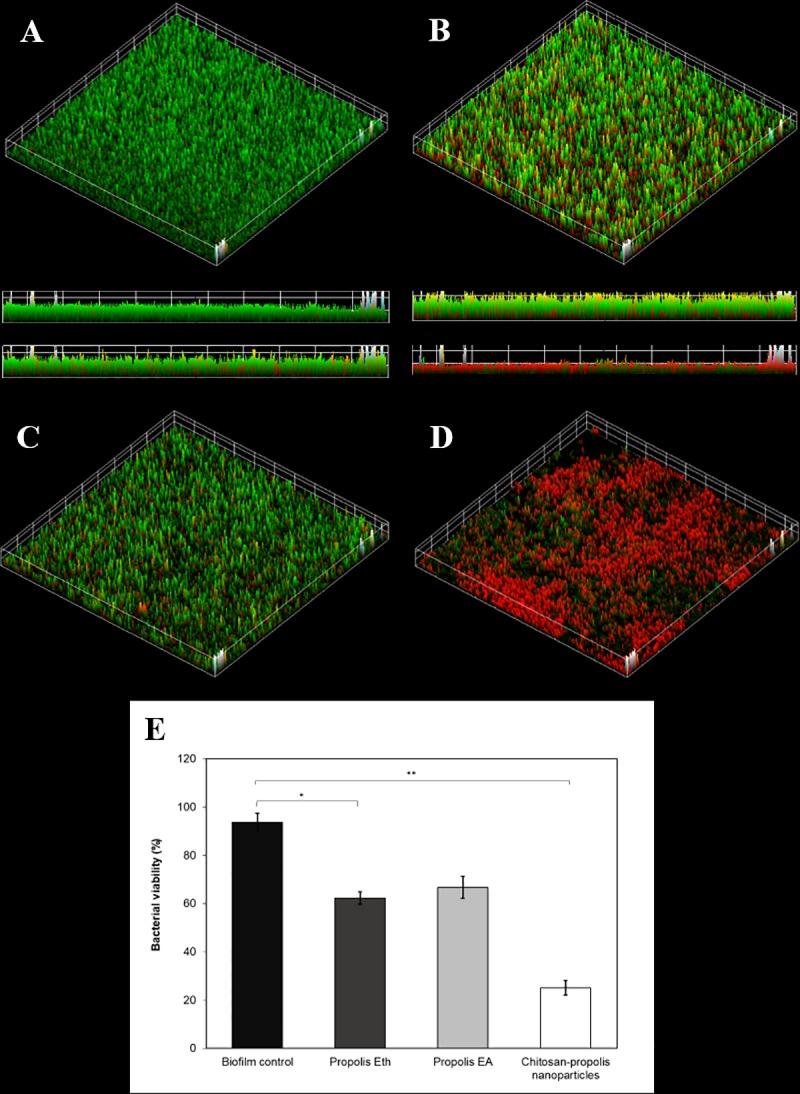
Imaging of live/dead biofilm bacteria using confocal laser scanning microscopy treated with propolis extracts and chitosan-propolis nanoparticles. Confocal laser scanning microscopy images depicting viability of *Staphylococcus epidermidis* biofilms bacteria. (A) Untreated biofilm control, (B) biofilm treated with Eth, (C) EA extracts of propolis and (D) chitosan-propolis nanoparticles. Bacterial viability in control versus propolis extracts or chitosan-propolis nanoparticles treatments is represented as graph (E). (*p <0.05; **p <0.01 compared to control group). Abbreviations: Eth: ethanol; EA: ethyl acetate.

The confocal laser scanning microscopy analysis (Fig 2) depicts live bacteria with intact membrane in green (SYTO9 green-fluorescent nucleic acid stain) whereas, non-viable bacteria with damaged membrane kinetics incorporate propidium iodide (red-fluorescent nucleic acid stain) and are stained red. In the control group (Fig 2A), untreated bacteria were stained green, indicating high viability (>90%) and uninhibited biofilm formation without membrane damage. In the presence of CPNP (Fig 2D), a large proportion of bacteria were stained red, indicating that CPNP treatment is effective in decreasing the viability of the bacteria (to ~25%) by causing membrane damage and also significantly reduced biofilm formation. Propolis Eth and EA extracts had only a marginal effect on biofilm formation as well as bacterial viability (Fig 2B and 2C) and the proportion of viable bacteria was ~60–70%. This confirms the anti-bacterial efficacy of chitosan-propolis nanoparticles.

### Propolis treatments affect survival of *S*. *epidermidis*

**Fig 3:**
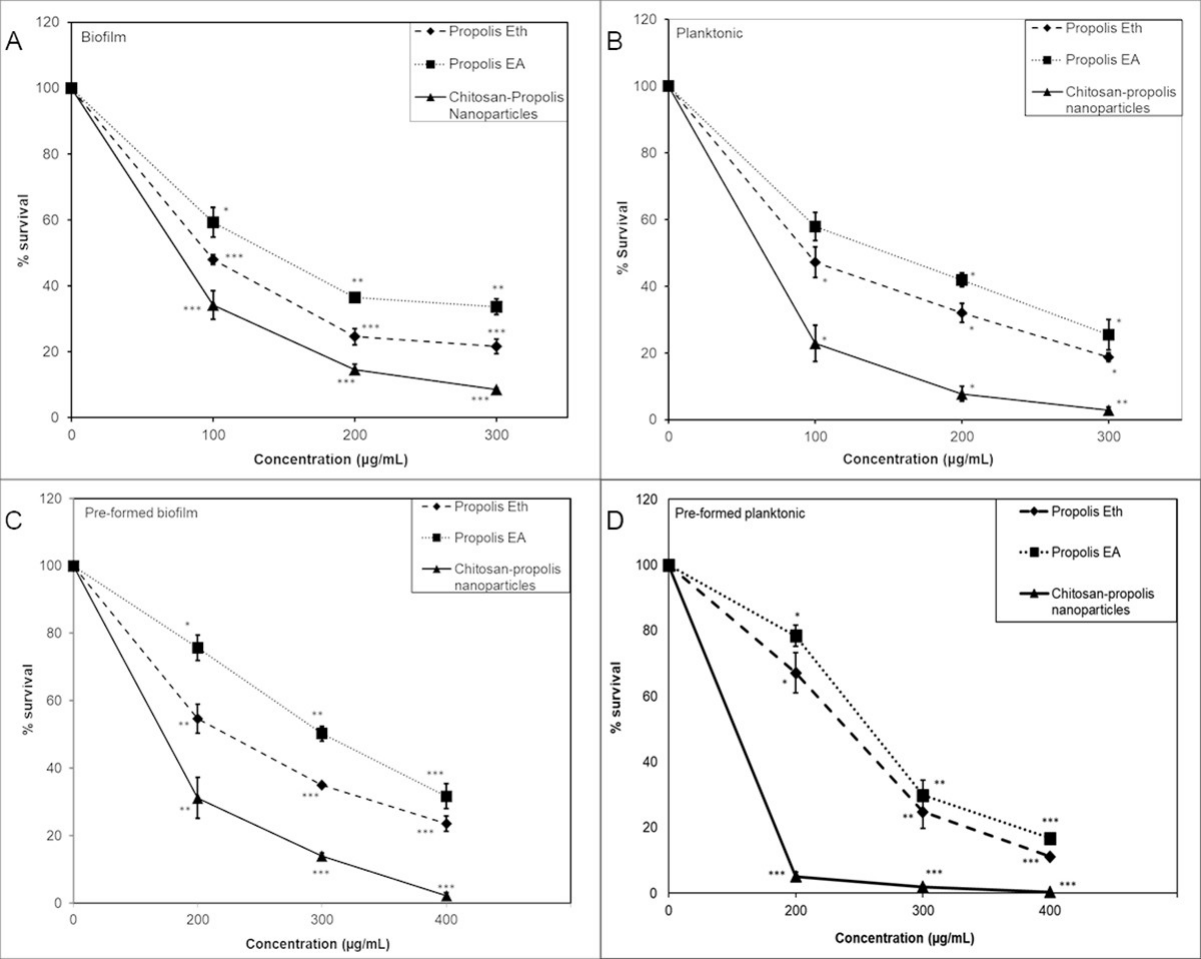
Survival of bacteria in biofilm and planktonic forms is affected by propolis treatments. Graphs representing the percentage survival of *S*. *epidermidis* bacteria present in biofilms (A and C) or in planktonic form (B and D) either co-treated (A and B) or treated as pre-formed biofilms (C and D) with propolis Eth or EA extracts or chitosan-propolis nanoparticles. Abbreviations: Eth: ethanol; EA: ethyl acetate.

The antibacterial efficacy of CPNP against the survival of *S*. *epidermidis* was evaluated and compared with that of propolis extracts against planktonic as well as biofilm bacteria. CPNP was found to be more efficient in decreasing the survival of *S*. *epidermidis* when compared to propolis Eth and EA extracts, especially in pre-formed biofilms (Fig 3). At 100 μg/mL concentration, CPNP decreased the survival of biofilm bacteria by ~70% compared to Eth or EA extracts where only 40%-50% decrease was observed (Fig 3A). When pre-formed biofilm was treated with CPNP (100 μg/mL), survival was decreased to ~60% whereas Eth or EA extracts managed to decrease survival only by 10% (Fig 3C). Planktonic bacteria also displayed greater sensitivity to CPNP treatment compared to Eth or EA treatment (Fig 3B and 3D). At higher concentrations, survival of bacteria was reduced to less than 20% with CPNP treatment. Overall, bacteria present in both biofilms as well as planktonic forms exhibited greater susceptibility to CPNP compared to ethanol or ethyl acetate extracts of propolis.

### Disruption of *S*. *epidermidis* biofilms by proplis treatments as imaged by scanning electron microscopy (SEM)

**Fig 4:**
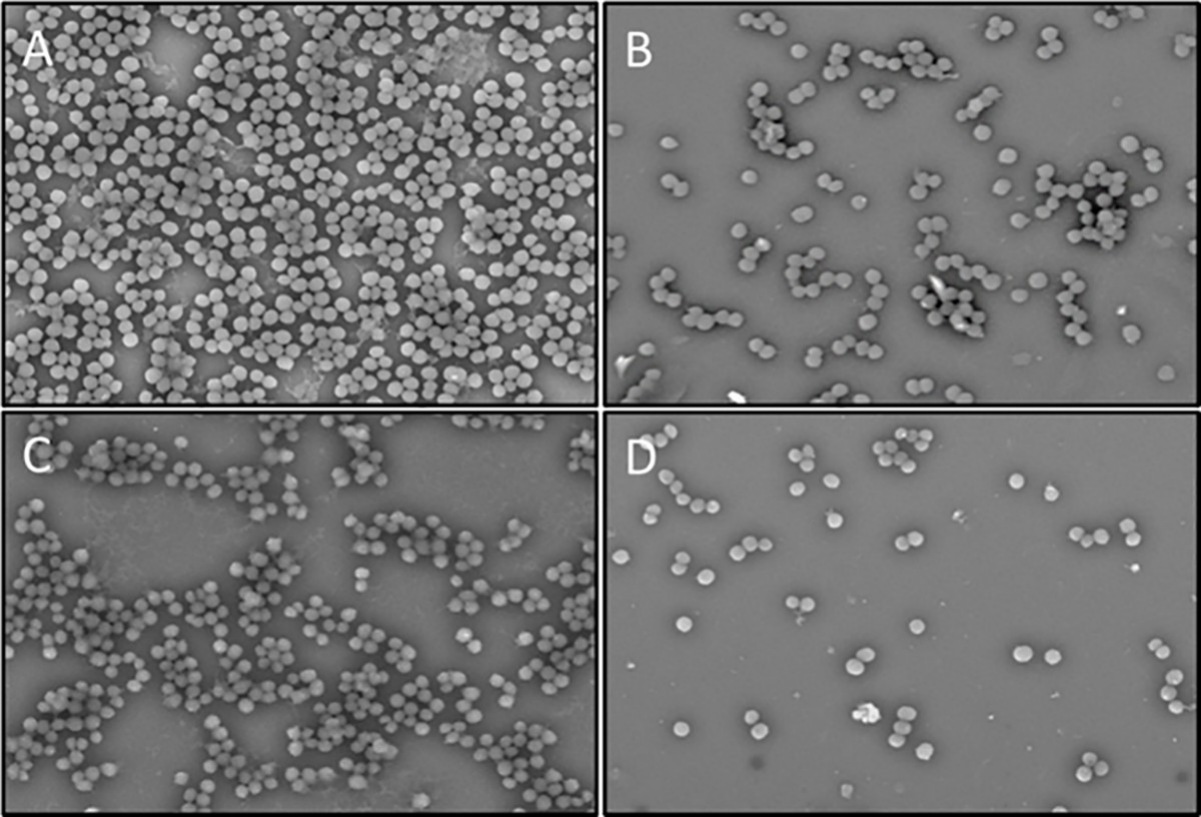
Disruption of bacterial biofilm by propolis treatments as imaged by scanning electron microscopy. SEM micrograph depicting bacterial biofilms of untreated control (A), biofilm treated with Eth (B) or EA (C) extracts of Malaysian propolis or chitosan-propolis nanoparticles (D). Abbreviations: Eth: ethanol; EA: ethyl acetate.

The morphology of bacterial biofilm was examined by scanning electron microscopy. The control samples showed a dense cluster of bacteria, which covered the entire surface attached to the matrix (Fig 4A). Bacterial number was reduced and biofilm was disrupted by treatment with Malaysian propolis extracts (Fig 4B and 4C). Treatment with CPNP on the other hand, resulted in disruption of biofilm with a significant decrease in bacterial numbers (Fig 4D). This data clearly showed that CPNP is more effective than propolis extracts in disrupting bacterial biofilms.

### Effect of propolis preparations on biofilm-related gene expression of *S*. *epidermidis*

**Fig 5:**
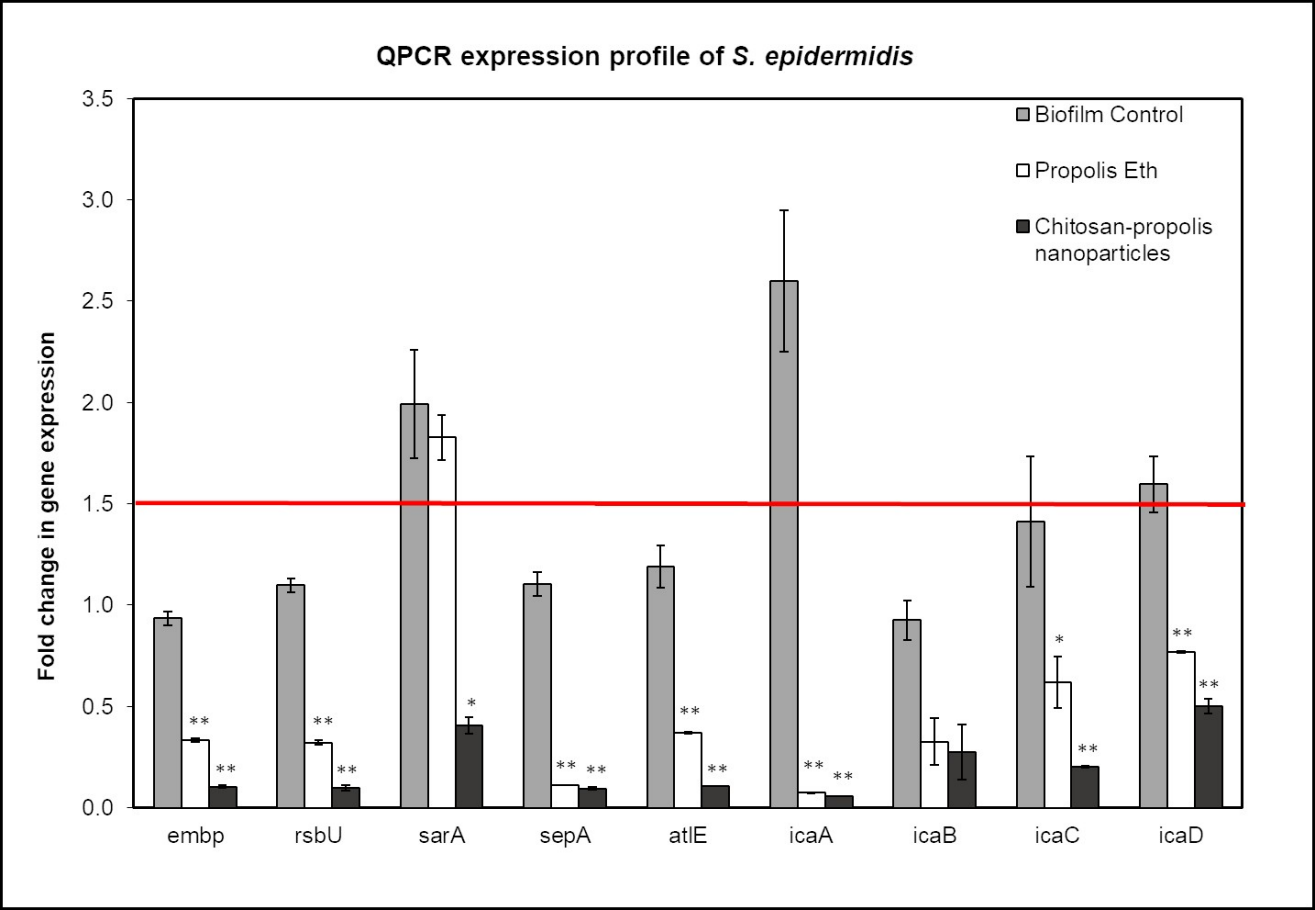
Real-time QPCR analysis of genes involved in biofilm formation of *S*. *epidermidis*. Relative expression of genes involved in biofilm formation of *Staphylococcus epidermidis* was determined by quantitative real-time PCR analysis. Total RNA was extracted from bacteria in different treatment groups (biofilm control, biofilm treated with propolis Eth extract and biofilm treated with chitosan-propolis nanoparticles), converted to cDNA and analyzed by qPCR using specific primers. (*p <0.05; **p <0.01 compared to control group). Abbreviation: Eth: ethanol.

Most of the genes analyzed showed increased expression in untreated control compared to the treated groups, but their expression was below 1.5-fold except for *sarA*, *icaA* and *icaD* genes (Fig 5). *IcaA* gene was significantly down regulated to ~0.1-fold when exposed to propolis extract and chitosan-propolis nanoparticles. *IcaBCD* genes were downregulated when treated with propolis Eth (~0.4–0.7-fold) and CPNP (~0.3–0.5-fold). *IcaABCD* genes encode proteins for the synthesis of polysaccharide intercellular adhesion (PIA) that is involved in intercellular adhesion of bacteria. The other genes (*Embp*—an intercellular adhesin, *sepA*—a metalloprotease, *altE*—autolysin E, *rsbU*—regulatory gene) were significantly down-regulated when treated with propolis Eth and even more when treated with CPNP. The regulatory gene sarA was only downregulated with CPNP treatment but not with propolis Eth treatment. These results show that treatment with CPNP is more effective than propolis extracts and all the treatments downregulate the genes involved in biofilm formation of *S*. *epidermidis*, causing inhibition of the same.

### Synergistic effect of chitosan-propolis nanoparticles with different antibiotics

Combinations of CPNP with the antibiotics- rifampicin, ciprofloxacin, vancomycin, doxycycline and gentamicin were tested in *S*. *epidermidis in vitro* biofilm model (Table 1). Synergism was observed between doxycycline and CPNP in inhibiting planktonic bacteria as well as biofilm growth. In case of pre-formed biofilms, synergism was observed between CPNP and the antibiotics- rifampicin (inhibits bacterial RNA polymerase), ciprofloxacin (inhibits DNA gyrase), vancomycin (inhibits cell wall synthesis in Gram-positive bacteria) and doxycycline (inhibits protein synthesis by binding to 30S subunit of bacterial ribosome). However, no synergism was found with gentamicin (inhibits protein synthesis by binding to 30S subunit of bacterial ribosome) and CPNP. These results suggest that CPNP can be useful as an adjunct in treating *S*. *epidermidis* infections.

**Table 1:** Summary of antibiotic synergy testing scores of *S*. *epidermidis* determined by checkerboard titration assays.

**A. Planktonic growth inhibition**						
	**Minimum inhibitory concentration (MIC) (μg/mL)**				**FICI**	**Synergy (≤0.5)**
	**Antibiotic alone**	**Antibiotic + CPNP**	**CPNP alone**	**CPNP + Antibiotic**		
Rifampicin	0.02	0.02	250	15.63	1.06	×
Ciprofloxacin	0.625	0.3125	250	15.63	0.56	×
Vancomycin	10	5	250	125	1	×
Doxycycline	200	25	250	31.25	0.375	✔
Gentamicin	0.78	0.39	250	125	1	×
**B. Biofilm inhibition**						
	**Minimum inhibitory concentration (MIC) (μg/mL)**				**FICI**	**Synergy (≤0.5)**
	**Antibiotic alone**	**Antibiotic + CPNP**	**CPNP alone**	**CPNP + Antibiotic**		
Rifampicin	0.02	0.02	250	15.63	1.06	×
Ciprofloxacin	0.625	0.3125	250	15.63	0.56	×
Vancomycin	10	5	250	125	1	×
Doxycycline	200	25	250	62.5	0.375	✔
Gentamicin	0.78	0.78	250	18.75	1.08	×
**C. Preformed biofilm**						
	**Minimum inhibitory concentration (MIC) (μg/mL)**				**FICI**	**Synergy (≤0.5)**
	**Antibiotic alone**	**Antibiotic + CPNP**	**CPNP alone**	**CPNP + Antibiotic**		
Rifampicin	0.16	0.04	500	125	0.5	✔
Ciprofloxacin	2.7	0.625	500	62.5	0.36	✔
Vancomycin	20	5	500	62.5	0.375	✔
Doxycycline	100	25	500	125	0.5	✔
Gentamicin	3.12	3.12	500	125	1.25	×

## Discussion

Net surface charge of bacteria is crucial for their survival, and alteration in the surface charge can have physiological consequences. Surface charge neutralization has been explored as an antibacterial activity employed by antimicrobial agents acting on bacterial surface. Zinc oxide nanoparticles with positive zeta potential were reported to have high antimicrobial activity against both Gram-positive and Gram-negative bacteria compared to those with negative zeta potential [20]. Exposure of *P*. *aeruginosa* to high concentrations of benzalkonium chloride, a cationic surfactant led to a reduction in the membrane negative charge caused by alteration in gene expression, thereby causing a major adaptative feature in the bacteria to withstand the surfactant effect [21]. Nanoparticles with positive surface charge are known to interact with bacteria with negative surface potential, thereby resulting in membrane depolarization and inhibition of bacterial growth [20]. The surface charges of antimicrobial agents also determine their binding efficacy. While propolis extracts lower the membrane potential of bacteria, our cationic nanoparticles have the opposing effect. They easily bind to the anionic bacteria and increase their zeta potential. Propolis extracts with a negative surface charge resulted in weaker interaction between the surfaces due to the repulsive force. Changes in zeta potential of bacteria affect their cell surface permeability; a change in zeta potential affects bacterial cellular physiology leading to mortality and/or inhibition of growth kinetics.

Biofilm structures are inaccessible to conventional antimicrobial agents. Sans-Serramitjana *et al*. validated the efficacy of nanostructured lipid carrier coupled with colistin in killing gram negative *Pseudomonas aeruginosa* biofilm using CLSM analysis and showed a significant reduction in biofilm viability after treatment [22]. The group proposed that the use of nanostructured carrier could help to deliver drugs and infiltrate into biofilm matrix efficiently, therefore capable to eradicate the living and dormant cells [22]. Cationic agents can potentially alter the zeta potential and are established as markers for the assessment of membrane damage in gram positive bacteria [18]. The ability to impair bacterial membranes is one of the properties envisaged in potential antibacterial drug targets, since this property demonstrated to reduce antibacterial resistance [23, 24].

Malaysian propolis and chitosan-propolis nanoparticles are reported to control biofilms formed by *Enterococcus faecalis* [17]. Planktonic bacteria can be eliminated relatively easily by antimicrobial agents or host immune responses whereas, biofilm bacteria show greater tolerance to treatments and can act as reservoirs of infection [25]. Our data corroborate the above findings as we found that lower concentrations are sufficient to kill planktonic bacteria compared to biofilm bacteria. Propolis extracts and CPNP are able to inhibit the growth of biofilm as well as to eliminate the pre-formed biofilm established by *S*. *epidermidis*. CPNP has better antibacterial efficacy as compared to propolis extracts. The differences may be attributed to the small particle size of chitosan-propolis nanoparticles, which enables them to penetrate into the biofilm and the positive zeta potential that lead to effective elimination of the bacteria.

Numerous studies have identified the factors that contribute to the development of *S*. *epidermidis* biofilm. To date, many genes that contribute to virulence and biofilm formation in *S*. *epidermidis* have been identified. We investigated *icaADBC*, *rsbU*, *sarA*, *sepA*, *embp* and *atlE* genes. Polysaccharides intercellular adhesion (PIA), or also known as poly-N-acetylglucosamine (PNAG) is one of the most studied functional molecules involved in biofilm development of *S*. *epidermidis*. PIA is synthesized by icaADBC-encoded proteins, and is also reported in *Staphylococcus aureus*, *Staphylococcus caprae* and *Escherichia coli* [26–29]. PIA facilitates biofilm formation as it promotes cell-cell adhesion. In addition, PIA also protects bacteria cells from host immune response [30] and mediates hemagglutination of erythrocytes [31, 32]. The *ica* genes are involved in initial adhesion and intracellular aggregation during biofilm formation by *S*. *epidermidis* [33]. Cafiso *et al*. reported that 45% of *S*. *epidermidis* isolated from hospitals carry *icaRADBC* genes and these isolates were more tolerant to antibiotics. They also found that at least two genes (*icaAD*) were up-regulated in biofilm-producing isolates of *S*. *epidermidis* [34].

PIA-, *aap*- or e*mbp*-mediated biofilm producing *S*. *epidermidis* strains are found to resist phagocytosis uptake by macrophages as well as countering activation of macrophage [35]. Embp, an extracellular matrix-binding protein mediates attachment of *S*. *epidermidis* to fibronectin and regulates biofilm formation [36]. However, Linnes *et al*. found that *embp* gene acts as a stress regulator against osmotic stresses, instead of facilitating attachment [37]. s*arA* and *rsbU* may be involved in the transcription of *ica* locus, which indirectly associated with the production of PIA for biofilm formation [38, 39]. Knocking down the expression of s*arA* gene in *S*. *epidermidis* diminishes the expression of *icaADBC* and compromises the ability to establish biofilms [39]. We found that both s*arA* and *icaA* were significantly up-regulated in biofilm bacteria. An extracellular metalloprotease gene product, SepA is capable of degrading antimicrobial peptides and helps in evading innate host responses [40]. The autolysin gene, *atlE*, possesses vitronectin-binding activity and is also capable of binding to polystyrene surface, suggesting that it played an important role in biomaterial-associated infections [41]. A combined downregulation of these biofilm-related genes indicates enhanced efficacy of CPNP treatment.

Propolis affects gene expression, decreases bacterial viability and interferes with biofilm formation of *S*. *epidermidis*, thereby rendering it sensitive to further treatment with antibiotics. This is evident from the synergistic effect of CPNP with selected antibiotics. Combination therapy with antibiotics + CPNP would help in decreasing the dose of antibiotics while disrupting biofilms to make the treatment highly effective.

## Supporting information

S1 Table List of primers used for quantitative PCR. (DOCX)
