# Supplementary material for: Cationic chitosan-propolis nanoparticles alter the zeta potential of S. epidermidis, inhibit biofilm formation by modulating gene expression and exhibit synergism with antibiotics
Source: PLoS One. 2019 Feb 28;14(2):e0213079. doi: 10.1371/journal.pone.0213079 (PMC6394969; doi:10.1371/journal.pone.0213079)
Supplement: S1 Table — (DOCX) [file pone.0213079.s001.docx]

**S1 table**

**S1 table. List of primers used for quantitative PCR.**

| **Gene** | **Forward Primer** | **Reverse Primer** |
| --- | --- | --- |
| ***rsbU*** | gcttatggacattcacaa | gattcatctcttcatacagt |
| ***sarA*** | gtaatgaacacgatgaaagaact | gcttctgtgatacggttgt |
| ***icaA*** | ctcttgcaggagcaatcaat | agagcacgtggttcgtactt |
| ***icaB*** | aatggcttaaagcacacgac | aaacaggaaaggcattgtca |
| ***icaC*** | tataggcgtcggaatgatgt | tccagttaggctggtattgg |
| ***icaD*** | gaggcaatatccaacggtaa | aaatttccgtgttttcaacatt |
| ***embp*** | aacttcaagatgcaaagacagatgc | aatgacgctttggctactgca |
| ***atlE*** | tgtcctgctttcacgtatga | tctttggaattggtgcattt |
| ***tpi*** | catctgataaaccttcgacagcttt | tgctatcttcaatcacggtatgaca |
